# An infant with hyperalertness, hyperkinesis, and failure to thrive: a rare diencephalic syndrome due to hypothalamic anaplastic astrocytoma

**DOI:** 10.1186/s12885-015-1626-x

**Published:** 2015-09-04

**Authors:** Alessia Stival, Maurizio Lucchesi, Silvia Farina, Anna Maria Buccoliero, Francesca Castiglione, Lorenzo Genitori, Maurizio de Martino, Iacopo Sardi

**Affiliations:** 1 Neuro-oncology Unit, Department of Paediatric Medicine, Anna Meyer Children’s University Hospital and Department of Health Sciences, University of Florence, Florence, Italy; 2 Pathology Unit, Anna Meyer Children’s University Hospital, Florence, Italy; 3 Neurosurgery Unit, Department of Neurosciences, Anna Meyer Children’s University Hospital, Florence, Italy

## Abstract

**Background:**

Diencephalic Syndrome is a rare clinical condition of failure to thrive despite a normal caloric intake, hyperalertness, hyperkinesis, and euphoria usually associated with low-grade hypothalamic astrocytomas.

**Case presentation:**

We reported an unusual case of diencephalic cachexia due to hypothalamic anaplastic astrocytoma (WHO-grade III). Baseline endocrine function evaluation was performed in this patient before surgery. After histological diagnosis, he enrolled to a chemotherapy program with sequential high-dose chemotherapy followed by hematopoietic stem cell rescue. The last MRI evaluation showed a good response. The patient is still alive with good visual function 21 months after starting chemotherapy.

**Conclusions:**

Diencephalic cachexia can rarely be due to high-grade hypothalamic astrocytoma. We suggest that a nutritional support with chemotherapy given to high doses without radiotherapy could be an effective strategy for treatment of a poor-prognosis disease.

## Background

Failure to thrive (FTT) is an important and relatively frequent problem in infancy. In developed countries, in presence of a FTT in childhood, an uncommon disease must be suspected if dietary and behavioral interventions have been unsuccessful. An insufficient caloric intake, inadequate caloric absorption, or increased energy requirements may cause a state of malnutrition and all the diagnostic efforts adopted should be aimed to distinguish the source [1]. Common pediatric conditions associated with FTT are gastro-esophageal reflux, Crohn’s disease, celiac disease, cystic fibrosis, psychiatric disorder like nervous anorexia and other chronic illness (neurological, cardiac, nephrological, rheumatological, oncological, pulmonary, immunological diseases and chronic infections). Although FTT of in pediatric age is frequent, the organic causes are rare [2].

Diencephalic Syndrome (DS), also known as Russell syndrome or diencephalic cachexia, is a rare condition associated with a hypothalamic/chiasmatic tumor [3]. The DS is a rare disorder but potentially lethal, therefore its rapid diagnosis and treatment are critical for patient’s life.

Tumors related with DS are usually pilocytic (WHO-grade I) or pilomyxoid (WHO-grade II) astrocytomas in young children. Clinical features of DS are loss of weight leading to severe emaciation despite a normal caloric intake, hyperalertness, hyperkinesis, and euphoria. Growth rate usually remains linear. Since the first report of DS, additional symptoms such as nystagmus, hydrocephalus and vomiting have been reported to be possible manifestations of the syndrome [4, 5].

Treatment of DS is strictly related to treatment of the hypothalamic lesion: it has been shown that DS clinical signs and symptoms regress when the tumor is surgically removed or reduced by non-surgical therapy. Since complete resection of hypothalamic-chiasmatic lesions is often difficult, partial resection is followed by chemotherapy and/or radiotherapy, which may represent the only treatment when the mass is not resectable [6]. However, being young children, radiotherapy is not the first choice of treatment. Various chemotherapy regimens were assessed for arresting tumor growth in this type of tumors. As long as the DS signs and symptoms are present, patients need nutritional support to avoid consequences of emaciation and cachexia.

The experience reported here suggests that high doses chemotherapy with autologous stem cell transplantation could be effective in achieving a lasting progression free survival in DS patients associated to a hypothalamic high-grade astrocytoma. The significance of our experience stems from the fact that there are only clinical studies for the treatment of low-grade gliomas in DS patients.

## Case presentation

**Fig. 1:**
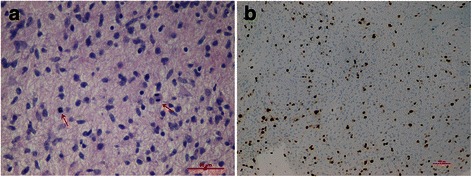
Photomicrographs of the lesion: astrocytes with nuclear atipia and mitosis (*arrows*) (**a**: hematoxylin and eosin, 40X) and high proliferation index (**b**, Ki-67 mmunocoloration, 10X). The surgical sample was routinely fixed in neutral buffered formol and embedded in paraffin. One 5 μm thick histological section obtained from each paraffin block was stained with hematoxylin and eosin. Further sections of the most representative paraffin block were used for immunohistochemistry and molecular analysis. Immunohistochemical studies were performed using the standard streptavidin-biotin technique and commercially available antibodies (Glial Fibrillary Acidic Protein, GFAP; p53 protein; Synaptophysin, SP; Ki-67). BRAF and H3F3 genes were analyzed as previously described [7, 8]

**Fig. 2:**
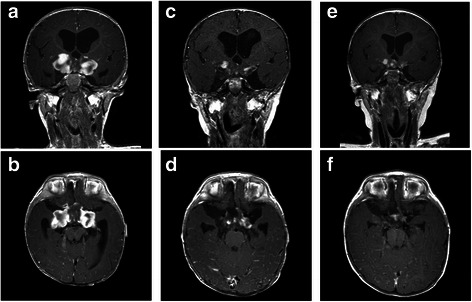
Serial MRI scans showing response to high doses chemotherapy in a five-month-old infant with hypothalamic anaplastic astrocytoma. Axial and coronal Gd-enhanced T1-weighted MR scans: immediately post-biopsy image of the hypothalamic lesion (**a**-**b**); **c** post-chemotherapy MRI scans demonstrating a partial response (**c**-**d**); MRI scans showing a stable disease 27 months from diagnosis (**e**-**f**)

In February 2013, a five-month-old infant was admitted to our hospital for weight loss (<3rd percentile) on the advice of his pediatrician (Fig. 1). He was a full-term newborn with no history of inadequate nutrition or gastrointestinal symptoms. The physical examination revealed pallid and dry skin, lack of subcutaneous fat, thin and triangular face, sunken anterior fontanelle, muscle wasting, restlessness, motor hyperactivity, euphoria, nystagmus and convergent strabismus of the left eye. Blood tests, analysis of stool samples and abdominal ultrasound excluded the malabsorption as cause of his state of malnutrition. A cranial TC scan was performed and revealed the presence of a lesion at the suprasellar region. The Gd-enhanced MRI of brain, subsequently required, showed contrast uptake in the perimedullary cisterns (Fig. 2,a-b). The spinal MRI showed absence of leptomeningeal metastases. The patient underwent to a biopsy with sub-frontal access and his postoperative course was without complications. The histopathological examination revealed a proliferation of astrocytes with nuclear atypia and mitotic activity (6X10 HPF) without vascular proliferation or necrosis. By immunohistochemical staining the neoplastic cells were GFAP positive and Synaptophysin and p53 negative. The proliferation index determined estimating the percentage of the Ki-67 positive neoplastic cells on the total of the tumor cells was about 18 %. We did not identify the V600E and KIAA1549 BRAF fusion gene mutations [7]. Furthermore, we did not evidence the presence of H3F3A K27M mutation [8]. The histopathological diagnosis was anaplastic astrocytoma (WHO-grade III). Diagnosis was confirmed by the review of the CNS national panel of pathologists. Therefore, he began a high dose chemotherapy program according to Italian schedule for Infant CNS tumors that included methotrexate, etoposide, cyclophosphamide, vincristine and carboplatin, two cycles of high-dose chemotherapy (thiotepa) and reinfusion of autologous stem cells [9]. The patient described in this study needed nutritional support and was monitored by the nutritional point of view during the treatment and the subsequent follow up. Parenteral nutrition was promptly started. Unfortunately the patient underwent several complications that hindered his weight recovery. He developed a syndrome of inappropriate secretion of antidiuretic hormone following the first two chemotherapy cycles. After only 1 month of parenteral nutrition, a central venous catheter infection by *Staphylococcus hominis* and *Staphylococcus epidermidis* occurred. Therefore, the parenteral nutrition was interrupted and replaced with enteral nutrition by nasogastric tube.

Five months after diagnosis, the MRI scans showed a lesion of reduced volume (partial response according to RANO criteria) [10] (Fig. 2,c-d). After recovery from the complications, weight started to increase until to a satisfactory level (Fig. 1). Our young patient was still being actively followed up at the time of this report. He remains alive with residual disease 27 months after starting chemotherapy (stable disease according to RANO criteria) [10] (Fig. 2,e-f).

## Conclusions

FTT can be defined as a condition characterized by a poor weight gain or severe weight loss. It may result from various disorders as unfavorable environments, psychological factors, or organic disease. The core question in the presence of suspected FTT is the cause of malnutrition. Diagnostic efforts should be directed toward determining whether the problem is secondary to inadequate intake, insufficient absorption, or excessive calorie requirement. Despite its clinical variability, FTT remains the most common symptom of DS at the diagnosis, and the main cause bringing the patient to medical attention, while length/height usually remains unaffected [4, 5, 11, 12]. Clinical features of emaciation, that may raise the suspicion of DS associated with CNS tumors, are weight loss, marked lack of subcutaneous fat and generalized lipodistrophy, without evidence of malabsorption, and evidence of psychic disturbance. Some of the most common symptoms at onset include visual acuity loss, visual field deficits, strabismus, nystagmus, proptosis and hypothalamic syndrome due to the hormonal dysfunction [4, 13–15].

It is not known whether weight loss in DS patients is due to increased energy consumption, inadequate absorption, or both. Neoplastic disease may lead to key changes of intermediary metabolism and feeding behavior, resulting in loss of fat reserves and lean body mass, i.e. the so-called cancer cachexia [16]. Supplemental feeding without any other intervention is not sufficient to reverse the syndrome, because emaciation and tumor growth go on. The mean survival of no-treated DS patients is usually less than 12 months [17]. Yet, effective therapy is hampered by the tumor location preventing its complete resection, and by significant neurocognitive and endocrinological sequelae associated with potentially curative radiotherapy. The role of surgery in treatment of optic chiasm lesions in DS patients is not clear. Surgery of hypothalamic area carries with it the risk of immediate, adverse neurologic and endocrinologic sequelae. The extensive surgical resection may result in a transient disease control. A recent report showed improvement of cachexia after partial surgery following failure of chemotherapy [7]. The positive impact of gross total removal on prognosis has been reported extensively in the literature. The Baby POG I study showed the degree of surgical resection had poor influence on overall survival as well as underlined in the French study on children under 5 years of age (BBSFOP) [18, 19].

In contrast to other LGG therapies, our recent experience, showed that lower doses of cisplatin (25 mg/m2/day) and etoposide (100 mg/m2/day) is probably one of the most active regimens with reduced neurotoxicity and myelotoxicity to treat children with DS related to low-grade optic-hypothalamic glioma [20].

The outcome for children with high-grade astrocytomas remains dismal despite aggressive surgical resection and radiotherapy followed by adjuvant chemotherapy. In an effort to improve the outcome for this group of children, clinicians have studied the role of high-dose marrow-ablative chemotherapy followed by autologous hematopoietic cell rescue.

Garrè et al. showed data on a small series of centrally reviewed malignant glioma in children younger than 3 years at diagnosis who were treated, in six instances, with a schedule of sequential high-dose chemotherapy utilizing high-dose methotrexate, etoposide, cyclophosphamide and carboplatin, followed by two courses with carboplatin/etoposide and thiotepa/melphalan at myeloablative doses and hematopoietic stem cell rescue. Focal radiotherapy was given only in case of progressive and/or residual disease at the end of chemotherapy [7].

We reported a rare case of diencephalic syndrome due to hypothalamic anaplastic astrocytoma (WHO-grade III). He enrolled to a chemotherapy program with sequential high-dose chemotherapy followed by two courses with thiotepa at myeloablative doses and autologous hematopoietic stem cell transplantation. The patient is still alive with stable disease and good visual function 27 months after starting chemotherapy.

Many investigators have evaluated the endocrinological abnormalities that might play a role in the pathogenesis of DS. It has been reported that either GH levels have been elevated or the regulation of GH secretion has been altered. Especially, partial GH resistance in DS patients has been observed [12]. Additional studies of hypothalamic-pituitary factors involved in appetite regulation and metabolism may clarify DS pathogenesis. It was hypothesized that cytokines as IL-1, IL-6, IFN-γ, TNF-α, brain-derived neurotrophic factor, MIC-1 may be involved in the cachexia process, in the imbalance which favors catabolism over anabolism, and in the neurologic and neuropsychiatric manifestations of the disease. Tumor-derived molecules, peptides/neuropeptides, neurotransmitters and hormones as leptin and GH-relin may interact with cytokines in the generation/development of the cachexia process [21]. Although these biochemical changes have been described as pathological mechanisms of the cancer cachexia, unfortunately biochemical causes of the DS remain unknown.

DS patients need an adequate nutritional support, which should be established as soon as possible. The oral route is the first choice. However, in several cases, enteral or parenteral nutrition may provide to children unable to feed the nutrients necessary to ensure proper growth, to correct or prevent malnutrition [22].

The choice between different types of artificial feeding should be done based on the clinical conditions. In particular, enteral administration of nutrients through the digestive system-wide probe is indicated in patients with oral nutrition not sufficient to maintain an adequate nutritional status. To this purpose, gastrostomy is widely applied for its safe, direct access to the stomach through the abdominal wall. Parenteral administration should be considered only as a second choice when enteral intervention has failed.

In this report we considered an anomalous clinical case of diencephalic cachexia due to an hypothalamic anaplastic astrocytoma. The winning strategy in the treatment of children with DS should take into account the nutritional support with chemotherapy given from standard to high doses, depending on the grade of the tumor. We suggest that a prompt diagnosis and a histological revision after diagnosis of hypothalamic tumors are mandatory for an accurate treatment of a poor-prognosis disease.

## Consent

Written informed consent was obtained from the legal guardians of the patient for publication of this Case report and any accompanying images. A copy of the written consent is available for review by the Editor of this journal.

## Abbreviations

FTT: Failure to thrive
DS: Diencephalic syndrome
CNS: Central nervous system
MRI: Magnetic resonance imaging
